# Clinical characteristics and associations of palmoplantar pustulosis: an observational study^☆^^☆☆^

**DOI:** 10.1016/j.abd.2019.04.008

**Published:** 2019-12-14

**Authors:** Ayse Oktem, Pınar Incel Uysal, Neslihan Akdoğan, Aslı Tokmak, Basak Yalcin

**Affiliations:** Department of Dermatology, Ankara Numune Training and Research Hospital, Ankara, Turkey

**Keywords:** Dermatitis, Dermatitis, allergic contact, Eccrine glands, Psoriasis

## Abstract

**Background:**

Palmoplantar pustulosis is a chronic and relapsing disease of the palms and soles, which is characterized by scattered clusters of pinhead-sized, sterile pustules.

**Objective:**

The aim of the present study was to determine demographic features, co-morbidities, and relation of palmoplantar pustulosis with psoriasis.

**Methods:**

A total of 48 patients (*M*/*F*: 15/33) were enrolled in the present study. A detailed history regarding age of onset, palmoplantar pustulosis duration, number of recurrences, personal and family history of psoriasis, accompanying arthritis, sternoclavicular tenderness, dental fillings, smoking status, and autoimmune disease was obtained; thorough dermatological examination was carried out. Patch testing results and laboratory investigations for thyroid autoimmunity were recorded.

**Results:**

Thirty-five of 48 patients (72.9%) were current smokers. Twenty of the 48 patients (41.7%) had dental fillings. There was not any significant correlation between palmoplantar pustulosis duration and dental filling duration (*p* = 0.170). Psoriasis was not detected in any patients either in history or in dermatological examination. Nail involvement and joint complaints were observed in seven of 48 patients (14%) and in nine of 48 patients (18%), respectively. Autoimmune thyroiditis was observed in four of 48 patients (12%). Patients with patch testing positivity (12.5% of patients, *M*/*F*: 1/5) had no considerable association for history of external contact with these materials.

**Study limitations:**

Retrospective analysis.

**Conclusion:**

Palmoplantar pustulosis appears to be a distinct entity from psoriasis. Routine thyroid functions test could be analyzed, but patch testing is not required in patients with palmoplantar pustulosis. Also, patients with palmoplantar pustulosis must be evaluated for musculoskeletal symptoms and signs.

## Introduction

**Figure 1:**
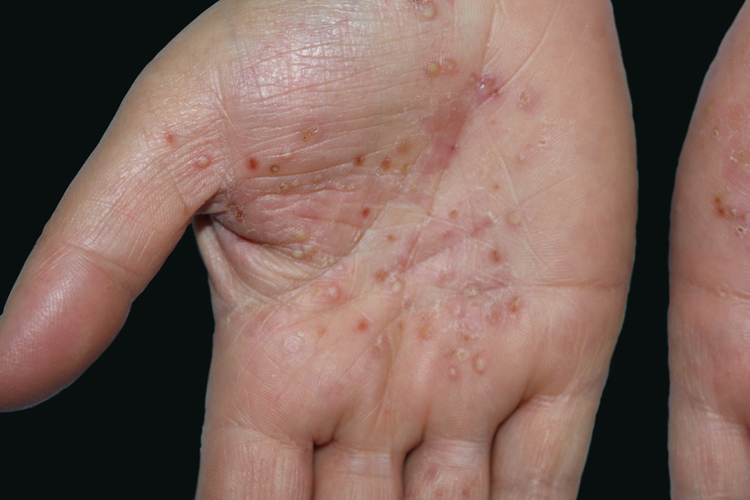
Pustules located on normal and erythematous skin of palms with brown discoloration.

Palmoplantar pustulosis (PPP) is a chronic and relapsing disease of the palms and soles, which is characterized by scattered clusters of pinhead-sized, sterile pustules, primarily located on the thenar, hypothenar, or central areas of the palms, either in a symmetrical or asymmetrical fashion (Fig. 1). Although initially thought to be a variant of psoriasis,^1^ PPP is now regarded as a separate entity rather than a disease on the psoriasis spectrum.^2^, ^3^ Despite the fact that some patients have psoriasiform plaques on other parts of the body, these lesions are not considered as the typical psoriatic plaques.^4^ Eccrine sweat glands and the acrosyringium play the central role in the pathogenesis of PPP.^5^, ^6^ PPP is a disease with female predominance. There is also a well-recognized association with smoking.^1^, ^7^ In addition, some reports notify that abnormality in thyroid functions^7^, ^8^ and contact sensitivity^9^ are associated with PPP. The aim of the present study was to determine demographic features, the frequency of thyroid autoimmunity, contact sensitivity, concomitant arthritis, nail involvement, and the presence of psoriatic lesions on dermatological examination in patients with PPP.

## Methods

A cross-sectional observational study was conducted. A total of 48 consecutive patients (15 men and 33 women) who were diagnosed with PPP between January 2014 and January 2018 and met inclusion criteria were enrolled in the present study.

The study was conducted according to the principles of the Declaration of Helsinki and approved by the local ethics committee. Written informed consent was obtained from all the participants before study inclusion. Study inclusion criteria were as follows: age ≥ 18 years and voluntarily agreeing to participate in the study. Occupational exposures of participants as well as demographical and clinical characteristics were recorded, including age of onset, duration of PPP, the number of recurrences, personal and family history of psoriasis, presence of psoriatic lesions at the time of data collection, accompanying arthritis and/or sternoclavicular joint tenderness, presence of dental filling, smoking habits, and autoimmune disease history. For each patient, a thorough dermatological examination was carried out. In particular, patients were carefully examined to detect concurrent psoriatic lesions in the rest of the body. Patch testing results and laboratory investigations for thyroid autoimmunity were recorded.

### Statistical analyses

Statistical analyses were performed with SPSS v. 21.0. Parametric variables are shown as means (mean ± SD), and nonparametric variables are presented as medians (median ± interquartile range [IQR]). Frequencies and percentages were calculated for the categorical variables. Statistical comparisons between normally distributed continuous variables and categorical variables were performed with Student's *t*-test. The Kolmogorov–Smirnov criterion was used for assessment of normality. To compare proportions (qualitative variables), the chi-squared test and the Fisher's exact test (in case expected values were < 5%) were employed. Mann–Whitney *U* and Kruskal–Wallis tests were used for comparing the non-normally distributed numeric variables. All *p*-values were two-sided and differences were considered statistically significant if *p*-values were < 0.05.

## Results

### Demographic and clinical characteristics

A total of 48 consecutive patients (15 men and 33 women; mean age, 50.9 ± 10.4 years [range: 21–65 years]) were enrolled in the present study. There was no significant association between men and women for the age of disease onset (*p* = 0.297). The median number of recurrences was ten in women, while it was two in men. The median disease duration was 36 months in women, while it was 12 months in men. The duration of PPP ranged from one to 360 months, and the number of recurrences ranged from one to 50 in the study group. The number of recurrences (*p* = 0.01) and the duration of the disease (*p* = 0.01) were significantly higher in women.

### Smoking habits

Thirty-five of 48 patients (72.9%) were current smokers and 13 of the patients (27.1%) were non-smokers. Among the non-smokers, 11 of 13 had never smoked, while two of 13 were ex-smokers. The ex-smokers were non-smokers for the last two to three years and had had smoking habits of 25 and 30 pack-years, respectively. The mean value of pack-years smoked was found to be 20 ± 13.3 (3–60 pack-years) in the study group. There was a statistically significant difference between men and women in smoking habits. The mean value of pack-years was significantly higher in males than females (*p* = 0.044).

### Dental filling

Twenty of the 48 of the patients (41.7%) had dental fillings. There was no significant difference between men and women in terms of dental filling duration. In addition, there was no significant correlation between PPP duration and dental filling duration (*p* = 0.170). Out of 11 patients who had dental fillings, two showed patch test positivity to nickel.

### Occupational exposure

Six of 48 patients reported occupational exposure. Occupations of these patients were as follows; cleaning staff (2), construction worker (2), seam worker (1), and hairdresser (1). Patch testing was positive in all patients with history of occupational exposure. Occupational exposure was higher in men than in women; however, the results did not reach statistical significance (26% *vs.* 6%, *p* = 0.067).

### Psoriasis in history and dermatological examination

Psoriasis was not detected in any patients, either in history or in dermatological examination. A family history of psoriasis or palmoplantar dermatitis was recorded in two of 48 patients (4.2%).

### Nail involvement

Nail involvement was observed in seven of 48 patients (14%). All patients with nail involvement had onycholysis; three of the seven also had pitting.

### Accompanying arthritis, sternoclavicular joint tenderness

Musculoskeletal complaints were observed in nine of 48 patients (18%). Statistical analysis revealed that there was no significant difference between men and women in terms of frequency of accompanying arthritis. Palpable tenderness of the sternoclavicular joint was reported by four of 48 patients (8.3%, *M*/*F*: 1/3). One of these patients had the history of ankylosing spondylitis. Since none of these patients exhibited typical manifestations such as pain, local swelling, or warmth,^10^, ^11^ imaging for radiographic changes was not performed. These patients were not considered to have any kind of sternoclavicular joint pathology, including hyperostosis.

Five patients with musculoskeletal complaints in the present study had already been evaluated by a rheumatologist for joint involvement. Two had a diagnosis of rheumatoid arthritis, two had diagnosis of ankylosing spondylitis, and one female patient was under follow up for seronegative arthritis with methotrexate therapy. Another four patients who did not previously seek medical care for their musculoskeletal complaints were seen by the rheumatology, orthopedic, and physical medicine and rehabilitation departments. One patient who suffered from knee pain had the diagnosis of discoid meniscus. Three patients had pain in the small joints of the hands, while one had pain only in the small joints of the left hand, and was finally diagnosed by the orthopedic surgeon as having cervical disk hernia. Another two patients, who had been evaluated by the rheumatology department, had no specific diagnosis. Regarding the relation between joint involvement and nail involvement, in total seven patients had onycholysis and three of them had arthralgia (one patient diagnosed with AS, one patient who had a diagnosis of seronegative arthropathy, and one patient with arthralgia in the small joints of hands with a nonspecific diagnosis).

### Autoimmune diseases

Autoimmune thyroiditis was observed in four of 48 patients (12%). All four patients were female. Other autoimmune diseases were observed in six (*M*/*F*: 1/5) of 48 patients (12.5%). Regarding the frequency of autoimmune diseases, including autoimmune thyroiditis, no statistically significant difference was established between male and female patients (*p* > 0.05).

### Spontaneous remission

Spontaneous remission was recorded in 19 of 48 patients (39.5%). Men and women were almost the same with respect to frequency of spontaneous remission (40% *vs.* 39%, *p* > 0.05).

### Patch testing results

Patch testing was positive in 12.5% of the subjects (one man, five women). Statistical analysis indicated that there was no significant difference between men and women in regard to patch testing positivity (*p* > 0.05). Patch testing positivity was seen in five patients for nickel, and one patient with concomitant positivity for Balsam of Peru and 2-methoxy-6-n-pentyl-4-benzoquinone. Patients with patch testing positivity had no considerable association for history of external contact with these materials. In two patients with nickel sensitivity, dental filling was present.

### Drug induced PPP

One patient was diagnosed with ankylosing spondylitis, in whom the lesions on the palms and soles were compatible with PPP following the etanercept therapy.

## Discussion

**Figure 2:**
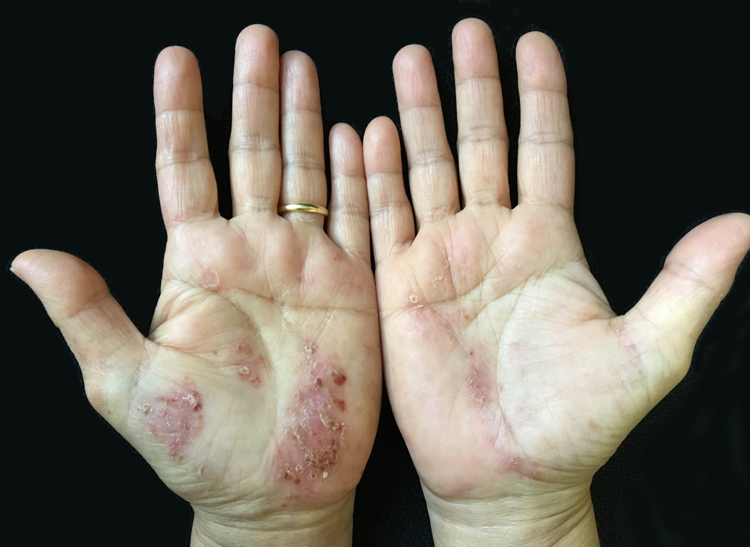
Desquamation and brown discoloration on erythematous skin of palms.

PPP affects mostly females and the age of disease onset ranges from 45 to 65.^1^, ^3^ Lesions are often accompanied by desquamation and brown discoloration during regression (Fig. 2). Recurrences and spontaneous remissions are common. Pustules can be located on normal or erythematous skin. Absence of any of psoriatic plaques, pustules located on areas other than palmoplantar region, and/or erythroderma or lack of personal/family history of psoriasis are the characteristic features of the disease. Patients who enrolled in the present study had neither classic plaque psoriasis lesions nor personal or family history of psoriasis. It has been suggested that PPP is an autoimmune disease triggered by nicotine^7^, ^8^; however, it is well known that smoking cessation does not always have a favorable effect on disease progression. In the present study, a significant ratio of patients had an active or previous history of smoking. However, 11 of 48 patients had no active or passive exposure to tobacco smoke.

Although PPP has been linked with psoriasis and psoriatic lesions located on body areas other than the palms and soles,^1^ it has been reported that these lesions actually were linked to psoriasis incorrectly; indeed, these psoriasiform lesions do not resemble classic psoriatic plaques.^4^ Currently, it is well known that psoriasis and PPP are almost separate entities with respect to cytokine profiles, clinical findings, and genetic susceptibility.^12^, ^13^

Nail involvement – including pitting, onycholysis, and dystrophy – can be detected in patients with PPP.^1^, ^14^ Seven of the present patients (14%) had onycholysis unrelated with onychomycosis; in additions, three of the seven also had pitting.

Smoking and thyroid gland dysfunction are the coexisting conditions most often investigated in patients diagnosed with PPP, and the association between tobacco use and PPP is well established.^1^, ^15^ The present study also found a considerable ratio of smoking, particularly in male patients. Thyroid gland dysfunction, including autoimmune thyroiditis, has been reported in many clinical studies.^1^, ^16^ The present study detected autoimmune thyroid disease in four of 48 patients as elevated anti-thyroid peroxidase antibodies, low levels of free-T4, and ultrasonographic findings of Hashimoto's thyroiditis.

Based on the report suggesting spontaneous regression of PPP after removal of dental amalgam,^9^ all patients were questioned for presence of dental fillings. There was no significant correlation between duration of PPP and duration of dental filling (*p* = 0.170). Out of the 11 patients who had dental filling, two patients showed patch test positivity to nickel sulfate. Based on these results, it is difficult to say that there is a contact sensitivity resulting from amalgam in the dental filling. Published reports indicate associations of PPP with arthralgia predominantly located in the hands and fingers,^16^ psoriatic arthropathy,^17^ and pustulotic arthro-osteitis (Sonozaki syndrome).^18^ However, some findings from these studies are debatable. For example, in the retrospective study of Becher et al.^17^ in which psoriatic arthropathy was reported in 12.3% of patients, a significant proportion of patients had family history of plaque psoriasis or concurrent plaque psoriasis. For this reason it is difficult to conclude there is a link between psoriatic arthropathy and PPP. Sternocostoclavicular hyperostosis (SCCH), which presents with swelling and pain of the sternoclavicular joint, was reported firstly in a Japanese patient with PPP. After this case report, Sonozaki termed this clinical entity as pustulotic arthro-osteitis (Sonozaki syndrome, PAO).^19^ Due to frequent association between PPP and pustulotic arthro-osteitis, they suggested that SCCH might be a musculoskeletal manifestation of PPP. In the present study none of the patients with PPP had clinical findings or history of SCCH.

It is well known that psoriatic nail disease, especially crumbling and onycholysis, are related with susceptibility to distal interphalangeal (DIP) arthritis in patients with psoriasis.^20^ One of the present patients who suffered from arthralgia in the small joints of the hands also had onycholysis, but she did not have objective clinical findings of DIP arthritis. In this context, the authors believe that nail and joint involvement should be evaluated among PPP patients in future studies with larger sample size.

Positivity of patch testing with allergens, especially with metals, was reported in PPP patients. In these case reports, positivity of patch testing is correlated with dental fillings including nickel, cobalt, and zinc.^9^, ^21^, ^22^ Removal of dental fillings led to the regression of PPP lesions in sporadic case reports.^9^ It is always important to relate patch testing results to history and clinical findings. It is difficult to say one of the etiopathogenic factors for PPP is systemic contact sensitivity because of the lack of large case-control studies. In the present series, two patients had both nickel positivity and dental fillings; however, the duration of dental filling was not compatible with PPP disease duration.

## Conclusion

Some findings of the present study – including female predominance, high prevalence of smoking habits, and nail involvement – are compatible with the literature. Based on the increasing evidence and the data presented here, PPP appears to be a distinct entity from psoriasis. Of note, the available studies clinically describing PPP are limited and controversial. The present study did not find a correlation between PPP and psoriasis. According to these results, the triggering role of contact sensitivity in the etiopathogenesis of PPP is unclear and patch testing may not be required in patients with PPP. Routine thyroid function tests could be analyzed in patients diagnosed with PPP due to the high prevalence of thyroid function abnormality in patients with PPP. Also, patients with PPP must be evaluated for musculoskeletal signs and symptoms. It is unclear whether typical PPP is associated with psoriatic arthritis or any other inflammatory arthropathies. In order to clarify this, arthritic symptoms should be investigated in large patients groups.

## Financial support

None declared.

## Authors’ contribution

Ayse Oktem: Approval of the final version of the manuscript; elaboration and writing of the manuscript; effective participation in research orientation; critical review of the literature.

Pınar Incel Uysal: Statistical analysis; effective participation in research orientation.

Neslihan Akdoğan: Conception and planning of the study; critical review of the literature.

Aslı Tokmak: Elaboration and writing of the manuscript; obtaining, analyzing, and interpreting the data.

Basak Yalcin: Approval of the final version of the manuscript; critical review of the manuscript.

## Conflicts of interest

None declared.
